# Maize Purple Plant Pigment Protects Against Fluoride-Induced Oxidative Damage of Liver and Kidney in Rats

**DOI:** 10.3390/ijerph110101020

**Published:** 2014-01-13

**Authors:** Zhuo Zhang, Bo Zhou, Hiaohong Wang, Fei Wang, Yingli Song, Shengnan Liu, Shuhua Xi

**Affiliations:** 1 Department of Occupational and Environmental Health, School of Public Health, China Medical University, No. 92 Bei Er Road, Heping District, Shenyang 110001, China; E-Mails: zhangzhuo618@139.com (Z.Z.); wangfei171@sohu.com (F.W.); songyingli860828@126.com (Y.S.); believeshun@163.com (S.L.); 2 Department of Nutrition and Food Hygiene, Shenyang Medical College, No146 Huanghe North Street, Shenyang 110034, China; E-Mails: zhoubo63@hotmail.com (B.Z.); wxh0515@hotmail.com (H.W.)

**Keywords:** fluoride, oxidative stress, anthocyanins, maize purple plant pigment, rats

## Abstract

Anthocyanins are polyphenols and well known for their biological antioxidative benefits. Maize purple plant pigment (MPPP) extracted and separated from maize purple plant is rich in anthocyanins. In the present study, MPPP was used to alleviate the adverse effects generated by fluoride on liver and kidney in rats. The results showed that the ultrastructure of the liver and kidney in fluoride treated rats displayed shrinkage of nuclear and cell volume, swollen mitochondria and endoplasmic reticulum and vacuols formation in the liver and kidney cells. MPPP significantly attenuated these fluoride-induced pathological changes. The MDA levels in serum and liver tissue of fluoride alone treated group were significantly higher than those of the control group (*p* < 0.05). The presence of 5 g/kg MPPP in the diet reduced the elevation of MDA levels in blood and liver, and increased the SOD and GSH-Px activities in kidney and GSH level in liver and kidney compared with the fluoride alone treated group (*p* < 0.05). In addition, MPPP alleviated the decrease of Bcl-2 protein expression and the increase of Bax protein expression induced by fluoride. This study demonstrated the protective role of MPPP against fluoride-induced oxidative stress in liver and kidney of rats.

## 1. Introduction

Fluoride is widely distributed in the natural environment and can lead to fluorosis due to excessive fluoride intake in many parts of the World. In addition to its well-known effects on the skeleton and teeth, fluorosis can also adversely affect soft tissues, such as the liver and kidneys [1,2]. The mechanisms by which fluoride produces such effects are still not clear. There is abundant literature reporting that fluoride increased the generation of reactive oxygen species (ROS) and free radicals, causes extensive oxidative stress and excessive lipid peroxidation, and reduces antioxidant enzyme activities *in vivo* or *in vitro* [3]. The oxidative stress has been considered an important mechanism of fluoride intoxication [4].

Fluoride can cross cell membranes by simple diffusion and enter soft tissues. The liver is one of the target organs attacked by fluoride. Numerous studies have revealed that excessive amounts of fluoride disturb the metabolic processes and detoxication capabilities of the liver [5]. Fluoride-induced necrosis, modifications of membrane lipids and apoptosis in hepatocytes [6], are associated with oxidative stress. The kidney has a prominent role in fluoride metabolism as 50%–80% of fluoride is removed via urinary excretion [7]. There was a close correlation between fluoride intake and renal injury. Fluoride-intoxicated rats showed increased ROS generation and lipid peroxidation in the kidneys [8]. In endemic fluorosis areas, drinking water fluoride levels over 2.0 mg/L can cause damage to the liver and kidney functions in children [9]. Fluorosis is irreversible, but can be prevented by appropriate intervention with antioxidants, such as quercetin, vitamins, phenolics and anthocyanins [10,11,12].

Anthocyanins, water soluble pigments found in plants, are polyphenols and well known for their biological antioxidative and anti-inflammatory benefits [13]. The antioxidative properties of anthocyanins arise from their high reactivity and ability to scavenge free radicals [14]. Maize is one of the most diverse grain crops found in Nature and one of the most widely cultivated cereals in the World. Purple maize is an important source of anthocyanins, phenolic compounds and carotenoids [15]. Maize purple plant anthocyanins have been reported to show antioxidant ability [16]. In the present study, maize purple plant pigment (MPPP) extracted and separated from maize purple plant was used for preventing or alleviating the adverse effects induced by fluoride in liver and kidney of rats, and the protective effects of MPPP were assessed.

## 2. Experimental Section

### 2.1. Chemicals and Reagents

Sodium fluoride (NaF, molecular weight 41.99) was procured from Sigma Chemical (St. Louis, MO, USA). Anti-Bax and anti-Bcl-2 antibodies were obtained from Santa Cruz Biotechnology (Santa Cruz, CA, USA). Glutathione (GSH), glutathione peroxidase (GSH-Px), superoxide dismutase (SOD), malondialdehyde (MDA) and total anti-oxidant capacity (T-AOC) test kits were purchased from NanJing Jiancheng Bioengineering Institute (Nanjing, China). All other analytical laboratory chemicals and reagents were obtained from Sigma, Invitrogen (Carlsbad, CA, USA) and Sangon Biotech Co., Ltd. (Shanghai, China). All chemicals and solvents were analytical grade. MPPP extracted and separated from maize purple plant was produced by Liaoning Dongya Seeds Co., Ltd (Shenyang, China). Zhou *et al.* [17] reported that MPPP used in this study contains 45.96% cyaniding-3-glucoside and 12.99% 3’,4’-dihydroxy anthocyanin-3-glucoside. MPPP was mixed in standard rodent diet, which was performed by the Shenyang Qianmin Animal Feeds Factory (Shenyang, China).

### 2.2. Animals and Treatment

Forty male and 40 female healthy weanling Wistar rats (60–80 g) obtained from Experimental Animal Center of China Medical University (Shenyang, China) were acclimated for a week before the start of the experiment and fed common basal pellet diet and water *ad libitum*. Rats were assigned randomly to four groups containing 20 rats per group: Group I: Control rats receiving unfluoridated water and common basal pellet diet. Group II: Experimental rats receiving drinking water containing 100 ppm fluoride and common basal pellet diet. Group III: Experimental rats receiving drinking water containing 100 ppm fluoride and diet mixed with 5 g/kg MPPP. Group IV: Experimental rats receiving drinking water containing 100 ppm fluoride and diet mixed with 10 g/kg MPPP. Although the fluoride concentration in drinking water cannot reach 100 ppm in endemic fluorosis areas, the fluoride dose used (100 ppm) in this study was selected with the following considerations in mind: (1) The rat fluorosis model needs to be established in order to observe the protective effects of MPPP against fluorosis. (2) Rats treated for 12 weeks with 100 ppm fluoride did not show any apparent signs of toxicity in our previous experiments. (3) Species differences exist between rats and human beings. 

During the course of treatment, daily water consumption, body weight gain and feed consumption were recorded periodically. All of the rats were kept in ventilated cages at 23–27 °C, with 55%–60% humidity and 12/12 h light/dark cycles. During the last exposure week, five rats per sex per group were placed in metabolic cages and 24 h urine samples were collected. After 12 weeks, exposure was stopped and rats were sacrificed under light ether anesthesia. Blood was collected from the abdominal aorta for the separation of serum. The livers and kidneys were removed immediately and weighed, and did not present any abnormality in appearance. The left external lobe of liver and cortex of left kidney were homogenized in chilled potassium chloride and centrifuged at 3,000 g for 10 min at 4 °C. The supernatant was used for biochemical analysis. Parts of middle lobe of liver and cortex of the right kidney were fixed in 2.5% glutaraldehyde for ultrastructure examination. All samples were stored at –80 °C until analysis. The experimental procedure used in this study met the guidelines of the Animal Care and Use Committee of China Medical University. 

### 2.3. Determination of Fluoride

The fluoride ion (F^−^) levels in serum and urine (10 rats each group) were measured potentiometrically directly after dilution with equal volumes of total ionic strength adjustment buffer using a fluoride ion-specific electrode and results were expressed as μg/mL. The concentrations of F^−^ in livers and kidney were determined according to the method of enzyme standard instrument-fluorine reagent colorimetric method [18]. Briefly, about 50 mg of tissue sample digested with lipase and protease was dissolved in an acid mixture (nitric acid and silver nitrate) in a closed compartment. The cover of the compartment was overlaid with saturated sodium hydroxide. After neutralization for 24 h, fluorine reagent was added into the mixture, and the values were calculated from a standard curve. The amount of fluoride was expressed in microgram of fluoride per kilogram of dry tissue.

### 2.4. Organo-Somatic Index

The body weight of each rat was recorded before killing. Liver and kidney were dissected out carefully, blotted free of blood and fresh weight was recorded. Organo-somatic index (OSI) was calculated by the following formula OSI = Weight of the organ / Weight of the body × 100.

### 2.5. Ultrastructure of Liver and Kidney

Liver and kidney tissues were fixed in a solution containing 2.5% glutaraldehyde in 0.1 M sodium cacodylate buffer at 4 °C, followed by rinsing in 0.1 M sodium cacodylate buffer. Subsequently, samples were postfixed in 1% osmium tetroxide, dehydrated through graded ethanol series, and embedded in Spurr’s resin. Resin sections of 50 nm were cut on resin microtome and ultrathin sections were stained with uranyl acetate and lead citrate, then observed and photographed using a HITACHI H-7650 (Hitachi Ltd., Tokyo, Japan) transmission electron microscope (TEM).

### 2.6. Determination of Serum and Tissue Oxidative Stress Markers

GSH levels and SOD activities of serum and tissues (20 rats each group) were measured with an improved 5,5’-dithiobis-(2-nitrobenzoic acid) (DTNB) method and hydroxylamine assay, respectively, provided by commercial test kits. GSH-Px activity was determined following the commercial test kit method, based on the reaction between glutathione remaining after the action of GPX and 5,5-dithiobis (2-nitrobenzoic acid) to form a complex that absorbed maximally at 412 nm. T-AOC activity was measured using a commercial kit, which was based on the production of stable color of the Fe^2+^-O-phenanthroline complex. The LPO was assessed by measuring MDA level. The quantification was based on measuring formation of thiobarbituric acid reactive substances (TBARS) according to the manufacturer’s protocol. Protein concentrations of tissues were determined by the method of Bradford [19] to normalize the levels of GSH, MDA and SOD activity. The results were expressed as mg/L for GSH, U/ml for SOD, GSH-Px and T-AOC, and nmol/ml for MDA in serum, and as mg/g protein for GSH, U/mg protein for SOD and GSH-Px, and nmol/mg protein for MDA in liver and kidney tissues.

### 2.7. Determination of Bax and Bcl-2 Protein Expressions in Liver and Kidney of Rats by Western Blot Analysis

Frozen liver and kidney tissue samples (five rats from each group) weighing 0.2 to 0.5 mg were cut into shivers with scissors first and washed with cold saline to remove blood. Tissue samples were lysed in ice-cold lysis buffer by supersound, and then centrifuged at 12,000 g for 25 min at 4 °C. Total protein content in each sample was quantified using the protein assay kit. Lysates with 30 μg protein each were subjected to electrophoresis on 10% SDS-polyacrylamide gel electrophoresis and transferred to a polyvinylidene difluoride (PVDF) membrane (Millipore, Bedford, MA, USA) at 12 volt 40 min. After the membranes were blocked in 5% fat-free dry milk in Tween 20 Tris-buffered saline (TBST) for 1.5 h, the membranes were incubated with primary antibodies at a dilution of 1:100 anti- Bcl-2, 1:1,000 anti- Bax and 1:1,000 polyclonal antibodies to β-actin, overnight at 4 °C. After washed with TBST, the membranes were incubated in horseradish peroxidase-conjugated secondary antibody (1:6,000) for 1 h and washed with TBST three times. Protein bands were detected by enhanced chemiluminescence reagent, and then densitometric analysis was performed with the Gel-Pro Analyzer version 3.0 software.

### 2.8. Statistical Analysis

The data were analyzed using the SPSS 13.0 software. The results are reported as means ± standard error (SEM). Differences between treatment groups were analyzed by one-way analysis of variance (ANOVA) followed by Dunnett’s test to compare means between the different treatment groups. A *p* < 0.05 was considered significant.

## 3. Results

### 3.1. Distribution of Fluoride in Blood, Urine, Liver and Kidney

Due to the changes of water and feed consumption during rat growth, the real fluoride intake or the real MPPP intake of rats changed with the amount of water and feed consumed. According to the daily water consumption and feed consumption of rats during the course of treatment, the real average amount of MPPP intake of rats was 0.045 g MPPP/100 g body weight/day for the group of diet mixed with 5 g/kg MPPP and 0.094 g MPPP/100 g body weight/day for the group of diet mixed with 10 g/kg MPPP. The average amount of real fluoride intake of rats was 1.699 mg F/100 g body weight/day. The fluoride levels of blood, urine, liver and kidney in 100 ppm fluoride treated rats were increased significantly compared with the non-fluoride-treated group (group I), but differences were not seen between the fluoride only treated group (group II) and fluoride-mixed MPPP treated groups (group III and IV). Treatment with MPPP at the both dose levels (5 g/kg and 10 g/kg) did not alter the fluoride levels of blood, urine, liver and kidney in 100 ppm fluoride -treated rats (Table 1).

**Table 1:** The levels of fluoride in serum, urine, liver and kidney of rats (X ± SEM).

Groups	N	Serum (μg/mL)	Urine (μg/mL)	Liver (μg/kg)	Kidney (μg/kg)
Group I	10	0.098 ± 0.007	3.501 ± 0.404	198.20 ± 37.15	284.53 ± 44.96
Group II	10	0.391 ± 0.014 *	26.559 ± 2.887 *	581.03 ± 64.63 *	914.32 ± 125.26 *
Group III	10	0.370 ± 0.031 *	26.014 ± 1.986 *	611.08 ± 81.03 *	923.42 ± 107.86 *
Group IV	10	0.378 ± 0.027 *	23.603 ± 2.018 *	509.67 ± 75.47 *	882.83 ± 83.55 *

Note: * *p* < 0.05 compared to the control group (Group I).

### 3.2. Organo-Somatic Index (OSI) of Liver and Kidney

There was no significant difference in body weight (weight gain) recorded between the control and treatment groups (data not shown). No significant change in the OSI of liver of any treatment group was observed as compared with the control group. A significant decrease (*p* < 0.05) in the OSI of kidney was found in fluoride alone treated rats (group II). The treatment of fluoride along with MPPP significantly attenuated the decrease induced by fluoride in the OSI of kidney (*p* < 0.05), and the OSI of kidney of Group IV was higher than that of Group II (*p* < 0.05), see Table 2. A protective effect of MPPP against the decrease of OSI of kidney caused by fluoride can be concluded. 

**Table 2:** Organo-somatic index (OSI) of liver and kidney of rats(X ± SEM, g/100 g).

Groups	N	OSI of Liver	OSI of Two Kidneys
Group I	20	2.58 ± 0.05	0.72 ± 0.01
Group II	20	2.61 ± 0.04	0.68 ± 0.01 *
Group III	20	2.65 ± 0.05	0.70 ± 0.01
Group IV	20	2.61 ± 0.03	0.72 ± 0.01 ^#^

Note: * *p* < 0.05 compared to the control group (Group I), # *p* < 0.05 compared to the Group II.

### 3.3. Ultrastructure Observation of Liver and Kidney

**Figure 1:**
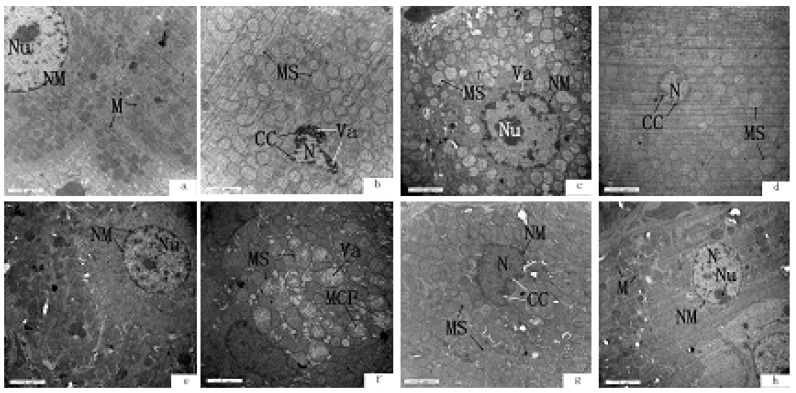
The ultrastructure of the liver and kidney tissues in rats. (**a**) liver of control rat; (**b**) liver of rat exposed to 100 mg/L fluoride; (**c**) liver of rat of exposed to 100 mg/L fluoride plus 5 g/kg MPPP; (**d**) liver of rat exposed to 100 mg/L fluoride plus 10 g/kg MPPP; (**e**) kidney of control rat; (**f**) kidney of rat exposed to 100 mg/L fluoride; (**g**) kidney of rat of exposed to 100 mg/L fluoride plus 5 g/kg MPPP; (**h**) kidney of rat exposed to 100 mg/L fluoride plus 10 g/kg MPPP.

Note: N, nucleus; Nu, nucleolus; NM, nuclear membrane; M, mitochondria; MS, mitochondria swelling; Va, vacuoles; CC, chromatin condensation; MCF, mitochondrial crest fracture; Magnification: 10000×.

Transmission electron microscope (TEM) analysis of the ultrastructure of liver and kidney in rats is shown in Figure 1. In the control group, liver and kidney exhibited normal integral structures, and the liver cells showed one or more oval nuclei with visible clear nucleoli and double nuclear membranes, abundant mitochondria, endoplasmic reticulum, and ribosomes in the cytoplasm of the liver cells (Figure 1a). 

In fluoride alone treated rats, liver cells showed cytomorphosis, vague cell boundaries, shrinkage of nuclear and cell volume, swollen mitochondria, broken or disappeared ridges, swollen endoplasmic reticulum, and apoptotic bodies (Figure 1b). In the rats treated with fluoride and MPPP (groups III and IV), liver cells still showed swollen mitochondria, but the numbers of cells with swollen mitochondria was decreased compared to group II, and pathological nuclear changes were attenuated (Figure 1c,d). The kidney cells in the control group were nearly round, with regular shape and clear cell boundaries, and the nucleus, mostly round or oval, was located in the center of the cells (Figure 1e). Kidney proximal tubule cells of fluoride-treated rats showed swollen mitochondria, vague or disappeared ridges, vacuole formation, and cell nuclei were pushed towards the basement membrane (Figure 1f). The swollen mitochondria of kidney proximal tubule cells in the fluoride plus MPPP groups showed a moderate decrease compared with the fluoride alone group (Figure 1g,h).

### 3.4. Lipid Peroxidation Levels and Antioxidative Status in Blood, Liver and Kidney of Rats

**Figure 2:**
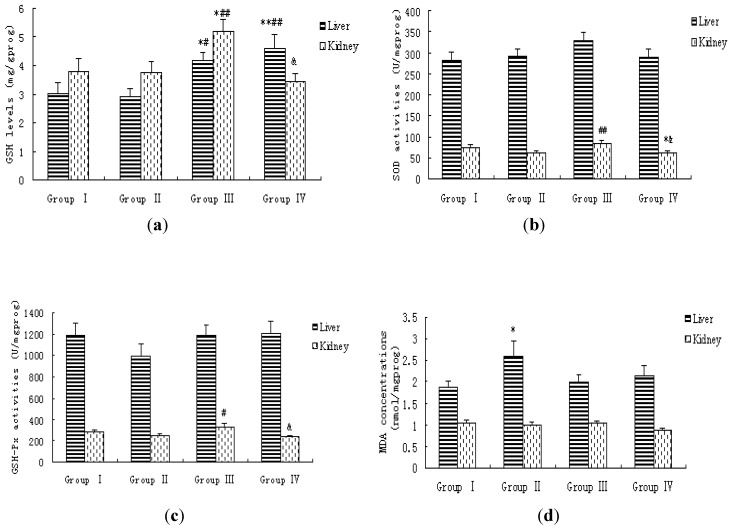
Lipid peroxidation levels and antioxidative status in liver and kidney of rats. (**a**) GSH levels; (**b**) SOD activities; (**c**) GSH-Px activities; (**d**) LPO production (MDA). Bars were presented as mean ± SEM.

Note: * *p* < 0.05 and ** *p* < 0.01 compared to the control group; # *p* < 0.05 and ## *p* < 0.01 compared to the only 100 ppm fluoride treated group (Group II); & *p* < 0.01 compared to the 100 ppm fluoride and 5 g/kg MPPP co-treated group (Group III).

In the fluoride alone treated group, the MDA levels in serum and liver tissue were significantly higher than those of the control group (*p* < 0.05). The presence of 5 g/kg MPPP reduced the elevation of MDA levels in blood and liver of fluoride alone -treated rats. No statistically significant changes in MDA levels in kidney tissue were observed for all groups (Table 3, Figure 2). 

**Table 3:** Oxidative stress markers in blood of rats (X ± SEM).

Groups	N	T-AOC	T-SOD	GSH	GSH-Px	MDA
		(U/mL)	(U/mL)	(mg/L)	(U/mL)	(nmol/mL)
Group I	20	6.64 ± 0.31	133.72 ± 3.67	12.99 ± 1.08	1311.27 ± 54.39	3.34 ± 0.24
Group II	20	6.54 ± 0.30	133.01 ± 4.03	13.34 ± 0.98	1295.68 ± 53.85	4.27 ± 0.37 *
Group III	20	6.55 ± 0.51	136.20 ± 4.92	11.44 ± 0.96	1462.25 ± 76.25 ^#^	3.19 ± 0.18 ^##^
Group IV	20	7.02 ± 0.35	139.92 ± 4.62	14.17 ± 1.59	1230.43 ± 47.24 ^&^	3.53 ± 0.34

Note: * *p* < 0.05 compared to the control group (Group I), # *p* < 0.05 and ## *p* < 0.01 compared to the Group II, & *p* < 0.01 compared to the Group III.

The antioxidative status was assessed by measuring the activities of SOD and GSH-Px, and GSH levels in blood, liver and kidney, as well as T-AOC activity of blood. The results showed that there were no significant differences for SOD activities in blood and liver among groups. SOD activity in kidney of rats co-treated with 5 g/kg MPPP and fluoride was significant higher than that of fluoride alone -treated rats (*p* < 0.01). However, SOD activity in kidney of rats co-treated with 10 g/kg MPPP and fluoride decreased and was significant lower than that of control group and fluoride plus 5 g/kg MPPP group. The GSH-Px activities in the blood and kidney were significantly increased in the fluoride plus 5 g/kg MPPP group compared with the fluoride alone-treated group, and decreased in the fluoride plus 10 g/kg MPPP group compared with the fluoride plus 5 g/kg MPPP group (*p* < 0.01). No significant changes were observed in liver for GSH-Px activity among the groups. The increases of GSH levels in liver and kidney are shown in Figure 2. The opposite was observed in the 5 g/kg MPPP and fluoride co-treated groups. Lower dose (5 g/kg) MPPP induced a significant increase in GSH levels compared to the control group and fluoride alone-treated group (*p* < 0.05) in liver and kidney. Higher dose (10 g/kg) MPPP also increased GSH levels compared with the control group and the fluoride alone-treated group in liver (*p* < 0.01), but this was not found in kidney. There were no significant changes in blood GSH level and T-AOC activity among all groups (*p* > 0.05).

### 3.5. Bax and Bcl-2 Expressions in Liver and Kidney of Rats

**Figure 3:**
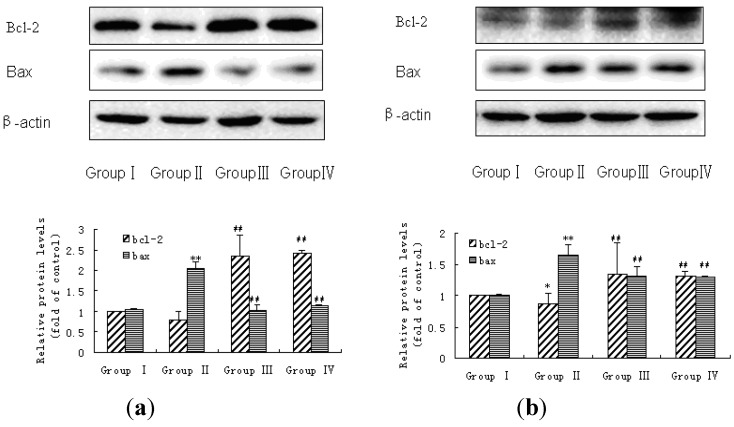
The expressions of Bcl-2 and Bax in liver and kidney of rats. (**a**) Liver was isolated for western blot analysis to determine protein expressions of Bcl-2 and Bax. (**b**) The expressions of Bcl-2 and Bax in kidney were determined.

Notes: The representative western blot was showed in upper panel. The panel below was a graphical representation of the relative densitometry of these bands. Loading was normalized to β-actin. Bars were presented as mean ± SD. * *p* < 0.05 compared to Group I, ** *p* < 0.01 compared to Group I, ## *p* < 0.01 compared to Group II.

Expressions of Bax and Bcl-2 in liver and kidney were measured by western blot analysis, as shown in Figure 3. The protein expressions of Bax in liver and kidney dramatically increased in fluoride alone-treated rats compares with the control group rats, and decreased in 5 g/kg and 10 g/kg MPPP groups compared with fluoride alone-treated group. 

On the contrary, Bcl-2 protein expressions of liver and kidney in the fluoride alone-treated group decreased significantly compared with the control group rats, whereas Bcl-2 protein of liver in 5 g/kg and 10 g/kg MPPP groups was elevated compared with the fluoride alone-treated group.

## 4. Discussion

A number of studies have reported that fluoride consumption leads to excessive formation of free radicals and lipid peroxidation in some organs and tissues [20]. Fluoride can increase LPO in blood and tissue of experimental animals [21]. Children suffering from endemic skeletal fluorosis show increased oxidative stress in terms of elevated MDA levels [22]. MDA is an important product of membrane lipid peroxidation and as a marker of oxidative stress in tissues. In this study, the MDA levels of serum and liver tissue in 100 ppm fluoride treated rats were significantly higher than those of the control group, which was consistent with the report of Shivarajashankara *et al.* [23]. Our results suggested that fluoride enhanced oxidative stress in rats. Oxidative stress is an imbalance between the production of free radicals and the body’s antioxidant defense system [24]. Antioxidant defense systems, including non-enzymatic and enzymatic antioxidant systems, can scavenge oxidative radicals from oxidative injuries. It was suggested that antioxidants (such as vitamins, methionine, N-acetyl-cysteine; polyphenolic flavonoids) and antioxidant-rich foods (such as blackberry juice) can act as antidotes for the management of fluorosis [25,26,27,28,29].

Anthocyanins, water soluble pigments found in plants, have health-promoting benefits including antioxidant and antiinflammatory effects [6,30]. The mechanisms of action of anthocyanins against a variety of oxidants include protecting cell membrane lipids from oxidation, protecting the amino acid tyrosine from the highly reactive oxidant peroxynitrite [31] and interfering with the dangerous hydroxyl radical-generating system [32]. Some reports have confirmed that anthocyanins are good antioxidants and can effectively eliminate free radicals [30]. Maize purple plant pigment, a natural antioxidant and rich in anthocyanins, showed antioxidative ability in the present study. We documented that the intake of 5 g/kg MPPP significantly reduced MDA levels in blood and liver of rats, increased SOD and GSH-Px activities in kidney and GSH levels in liver and kidney of rats. MPPP plays a crucial role in mitigation and/or protection against oxidative stress induced by fluoride through possibly directly scavenging and/or inhibiting ROS. The present study provides evidence of the antioxidant properties of MPPP to resist fluoride-induced oxidative stress due to the antioxidant actions of this compound.

Chronic fluorosis can severely damage many systems of the human body. ROS and lipid peroxidation have even been considered to play an important role in the pathogenesis of chronic fluoride toxicity and oxidative stress was as one of the important mechanisms of the toxic effects of fluoride. The liver is the main organ responsible for metabolism and detoxication. Fluoride exposure would induce both pathomorphological and metabolic changes in the liver [33]. In this study, histological changes of liver in rats treated with fluoride alone were characterized by cytomorphosis, vague cell boundaries, shrinkage of nuclear and cell volume, swollen mitochondria and endoplasmic reticulum and apoptotic bodies. Fluoride-treated kidney proximal tubule cells showed swollen, mitochondria, vague or disappeared ridges, and vacuole formation. Our histopathological findings confirmed that morphological changes of liver and kidney in fluoride-treated rats may be due to oxidative damage induced by the accumulation of fluoride in the liver and kidney. Similar observations were made by other authors who found cloudy swelling, hypertrophy, and atrophy of glomeruli in kidney of mice treated with fluoride [34]. These histological changes could be correlated with oxidative damage. The presence of MPPP alleviated the harmful effects of fluoride, such as mitigating pathomorphological changes of liver and kidney, increasing SOD and GSH-Px activities and decreasing MDA levels in liver and kidney. The antioxidant activities and health benefits of MPPP may be related to its high anthocyanins and phenolic content. The anthocyanins have shown a higher antioxidant activity than vitamins C and E. These compounds are able to capture free radicals by donation of phenolic hydrogen atoms [35].

ROS have been implicated as potential modulators of apoptosis and oxidative stress plays a role in apoptosis. The ability of oxidative stress to provoke apoptosis as a result of massive cellular damage has been associated with lipid peroxidation and protein and nuclei alterations. Previous studies showed that fluoride-induced apoptosis by oxidative stress and suggested the role of oxidative stress in the apoptotic process [36]. Necrosis of hepatocytes has also been observed in the presence of relatively high fluoride concentrations via increased oxidative stress [37]. Apoptosis is regulated by complex pro- and anti-apoptotic genes. Bcl-2 family member has been demonstrated to be involved in regulating apoptosis. Bcl-2 inhibits apoptosis and Bax promotes apoptosis, and they are widely present in the mitochondria, nuclear membrane, and endoplasmic reticulum. The mitochondrium is one of the most important organelles in apoptosis. Mitochondria swelling and rupture may release a large number of apoptosis-promoting factors. Bcl-2 expression was down-regulated in fluoride-treated human gingival fibroblasts [38]. A positive correlation was observed between the fluoride concentration of water and the expression of Bax in liver of fish after fluoride exposure [39]. Our results also showed that Bcl-2 protein expression decreased and Bax protein expression increased in fluoride treated liver and kidney of rats and MPPP can block Bcl-2 expression decrease and Bax expression increase. The present result supports the argument that oxidative stress plays an important role in apoptosis.

As expected, significant increases in F^−^ excretion in urine and F^−^ concentrations in serum, liver and kidney occurred following administration of fluoride in drinking water. Co-exposure to F and MPPP did not affect the concentrations of F^−^ in serum, liver and kidney and the rate of urinary F^−^ excretion. Also MPPP lacked any significant effect on urinary fluoride excretion. Our results may indicate that the protective effect of MPPP was because of its free radical scavenging and antioxidant activity, not by enhancing urinary fluoride excretion and decreasing the levels of fluoride.

However, owing to fluoride dose was used in this rat model was much higher than the fluoride concentration in drinking water in endemic fluorosis areas, there are the limitations to directly apply the results to the pathology associated to endemic fluorosis areas. 

## 5. Conclusions

The present findings suggest that high doses of fluoride have the potential to cause oxidative stress, which could provoke pathological changes, as well as cell apoptosis in the liver and kidney of rats. Anthocyanin-rich MPPP feeding could mitigate the fluoride-induced damage by elevating the antioxidant ability in the liver and kidney of rats. This study demonstrated the protective role of MPPP against fluoride-induced oxidative stress in liver and kidney of rats. MPPP might be an important antioxidant to apply in ameliorating fluorosis in the future.
